# Shells and Spheres: An *n*-Dimensional Framework for Medial-Based Image Segmentation

**DOI:** 10.1155/2010/980872

**Published:** 2010-06-10

**Authors:** Aaron Cois, John Galeotti, Robert Tamburo, Michael Sacks, George Stetten

**Affiliations:** ^1^749 Benedum Hall, University of Pittsburgh, Pittsburgh, PA 15261, USA; ^2^306 CNBIO, 300 Technology Drive, University of Pittsburgh, Pittsburgh, PA 15219, USA

## Abstract

We have developed a method for extracting anatomical shape models from *n*-dimensional images using an image analysis framework we call *Shells and Spheres*. This framework utilizes a set of spherical operators centered at each image pixel, grown to reach, but not cross, the nearest object boundary by incorporating “shells” of pixel intensity values while analyzing intensity mean, variance, and first-order moment. Pairs of spheres on opposite sides of putative boundaries are then analyzed to determine boundary *reflectance* which is used to further constrain sphere size, establishing a consensus as to boundary location. The centers of a subset of spheres identified as *medial* (touching at least two boundaries) are connected to identify the interior of a particular anatomical structure. For the automated 3D algorithm, the only manual interaction consists of tracing a single contour on a 2D slice to optimize parameters, and identifying an initial point within the target structure.

## 1. Introduction

The framework of Shells and Spheres described in this paper is based on a set of spheres called a *sphere map*. A sphere map consists of exactly one sphere centered at each image pixel, whose radius can be adjusted. Calculations denoted as *Variable-Scale Statistics *(VSSs) are performed on populations of pixels within spheres, as well as populations of adjacent and overlapping spheres. Memory and computational requirements are kept reasonable by storing only a relatively small, fixed number of VSSs at every pixel, many of which can be updated incrementally when growing or shrinking spheres. The ultimate goal of adjusting radii is to produce a sphere map in which each sphere is as large as possible without crossing a boundary. In the optimized sphere, the sphere map is thus equivalent to what is commonly known as a *distance map* [1], that is, the distance to the nearest boundary. Though the task is trivial with binary images, where definitive boundaries are known, it presents a challenge when boundaries are difficult to determine due to noise and tissue inhomogeneity. Our approach is well suited to this challenge, with the caveat that the correctness of segmentation of real images is generally subjective. 

Many conventional methods for image processing consider a region of fixed size and shape, usually referred to as a *kernel*, especially when used for convolution. Other common approaches define dynamic regions adjoining boundaries using deformable contours [2] or level sets [3]. Our approach, instead, uses a set of spheres whose individual radii are optimized using VSS operators to achieve maximum discrimination between adjoining image regions. Not only do such spheres provide highly representative populations for boundary detection, but those spheres that touch at least two boundaries are also *medial*, providing a basis for medial feature extraction. Unlike Gaussian blurring, commonly used in multiscale analysis [4], Shells and Spheres preserves sharp boundaries with increasing scale. This paper presents the notation and basic operators of Shells and Spheres for computing VSS. Using this framework, a wide variety of algorithms for sphere map optimization are possible, and we present two such algorithm, a trivial one for noiseless images and a practical one for real medial images with noise. We then extend this algorithm to include methods to identify boundary and medial locations, followed by an application of that extended algorithm to image segmentation. 

A primary goal of the research presented here is to extract medial ridges from images. The lineage of the medial approach may be traced to the medial axis (otherwise known as the symmetric axis or skeleton) introduced on binary images by Blum and developed [5–7]. Pizer extended the medial axis to gray-scale images, producing a graded measure called *medialness*, which links the aperture of the boundary measurement to the radius of the medial axis to produce what has been called a *core*. A core is a locus in a space whose coordinates are position, radius, and associated orientations [8, 9]. Methods involving these continuous loci of medial primitives have proven particularly robust against noise and variation in target shapes [10]. Models including discrete loci of medial primitives have also provided the framework for a class of active shape models known as *deformable m-reps* (sampled medial representations) [11], as well as a statistical approach using pairs of detected boundary points known as *core atoms* developed previously by Stetten and Pizer [12]. An excellent review of medial approaches and theory may be found in a recent text by Siddiqi and Pizer [13].

 The following sections will present the fundamental framework and basic operators of Shells and Spheres, as well as a detailed description of the two algorithms just mentioned. A summary of the experimental validation already published elsewhere is also included, and a brief discussion.

## 2. Shells and Spheres Framework

We begin by defining our notation. Shells and Spheres is inherently *n*-dimensional. For brevity, we use the term *sphere* regardless of image dimension, instead of the dimension-specific terms *disk (circle)*, *sphere*, or *hypersphere*. Figures are presented in 2D for ease of illustration.

Since the framework of Shells and Spheres is used to gather statistics on dynamic populations of pixels, we adopt a hybrid form of notation derived from standard set theory and statistics. We denote vectors by lowercase bold-faced letters (**x**), scalars by lowercase italic letters (*r*), and sets by uppercase letters (*S*). We use *ℤ* to denote the set of all integers, and *Ω* ⊂ *ℤ* ^*n*^ to denote the set of all pixel locations in a sampled *n*-dimensional image. 

 Given an *n*-dimensional image with intensities *f*(**x**) for **x** ∈ *Ω*, we define a *sphere map*, which assigns the radius *r*(**x**), the radius of a sphere centered at pixel **x**. We define a sphere to be an *n*-dimensional neighborhood of pixels that lie within a radius *r* of a center point. We use an integer value for *r*, such that a sphere of radius *r* centered at a pixel **x** is given by

(1) $$S_{r}\left(x\right)=\left\{y:\text{round}\left(\left|y-x\right|\right)\leq r,\;\;y\in \Omega \right\},$$

where *S*_*r*_(**x**) is a sphere of radius *r* centered at image pixel **x**, and **y** is a pixel within that sphere. Note the shorthand notation for the subscript *r*, meaning *r*(**x**), the radius of the particular sphere at **x** as given by the sphere map. In some instances, the reader will encounter an example with a different subscript, such as *S*_1_(**x**), meaning a sphere of radius 1, irrespective of *r*(**x**). By definition, **x** ∈ *S*_*r*_(**x**) for all **x**, even when *r*(**x**) = 0, and hence *S*_*r*_(**x**) is always nonempty.

A *shell* is a set of all pixels whose distance to the center rounds to a given radius, defined for radius *r* as

(2) $$H_{r}\left(x\right)=\left\{y:\text{round}\left(\left|y-x\right|\right)=r,\;\;y\in \Omega \right\}.$$

Shells are nonoverlapping such that for concentric shells,

(3) $$H_{p}\left(x\right)\cap H_{q}\left(x\right)=\emptyset ,\;p\neq q.$$

Additionally, shells are space filling, and thus a sphere of radius *r* may be formed from a union of shells,

(4) $$S_{r}\left(x\right)=\overset{r}{\underset{k=0}{⋃}}H_{k}\left(x\right).$$

**Figure 1:**
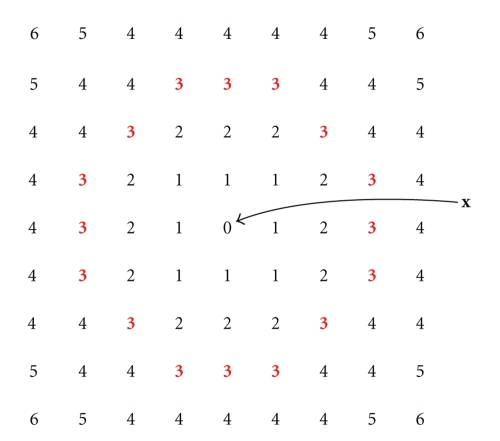
Each pixel is shown as a number indicating its integer distance from the central pixel. If we denote the central pixel as **x**, then pixels labeled *n* are members of the set *H*_*n*_(**x**). For example, the pixels labeled “3” (shown in bold) comprise the shell *H*_3_(**x**).

Figure 1 illustrates the distribution of pixels in a series of concentric shells surrounding a central pixel in a 2D image. Each pixel is labeled with its integer radius from the central pixel (labeled “**x**”). The shell *H*_3_(**x**) is shown as the pixels labeled “3” in bold. 

**Figure 2:**
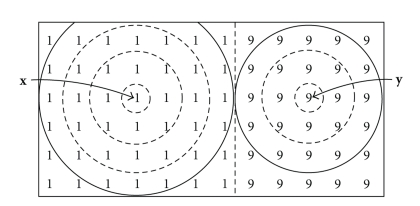
Noiseless image with boundary between two objects. Correctly scaled spheres *S*_*r*_(**x**) with *r*(**x**) = 3 and *S*_*r*_(**y**) with *r*(**y**) = 2 touch, but do not cross, the boundary. Numbers indicate pixel intensity.

Figure 2 shows an image containing two noiseless objects with pixel intensities of 1 and 9, respectively. Note that pixels in this case are represented by their intensity. The boundary between the image objects is identified by a straight dashed line. Pixel **x** is surrounded by a concentric set of four shells *H*_0_(**x**), *H*_1_(**x**), *H*_2_(**x**), and *H*_3_(**x**), shown separated by dashed circles. Shell *H*_3_(**x**) is truncated by the edge of the image. The union of all four shells is *S*_*r*_(**x**), shown enclosed by a solid circle, also truncated by the edge of the image, with a radius governed by the value of *r*(**x**) = 3 in the sphere map. Similarly, on the other side of the boundary, pixel **y** with an intensity value of 9 has three shells whose union *S*_*r*_(**y**) has a radius *r*(**y**) = 2. Both *S*_*r*_(**x**) and *S*_*r*_(**y**) touch but do not cross the boundary and are therefore correctly optimized.

**Figure 3:**
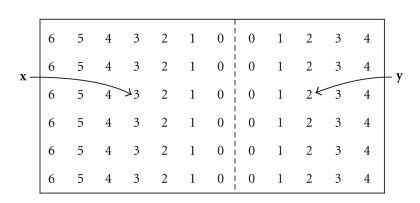
Correctly optimized sphere map of the image in Figure 2. Numbers indicate the integer radius of the sphere at each pixel. Pixels **x** and **y** are labeled as before.

The correctly optimized sphere map of the image in Figure 2 is shown in Figure 3, with each pixel represented by the radius of the sphere centered at that pixel. Note the linear increase in sphere radius with distance from the boundary and the fact that the radius equals zero adjacent to the boundary.

## 3. Variable Scale Statistics

We derive a number of statistics at pixel **x** (and every other pixel), calculated on the intensities of pixels within the sphere *S*_*r*_(**x**). Since these statistics depend on the radii of the spheres, we call them Variable Scale Statistics (VSS). We denote as *primary statistics* those VSS at **x** calculated using only the population of pixels within *S*_*r*_(**x**). Higher-order *secondary statistics* are VSS derived from multiple spheres.

## 4. Primary Statistics

The primary statistics at pixel **x** concern only the population of pixels within the sphere *S*_*r*_(**x**). Thus the *mean* at pixel **x** is the mean intensity of all pixels within the population *S*_*r*_(**x**) at its current radius, defined as

(5) $$\mu \left(x\right)=\frac{1}{\left|S_{r}\left(x\right)\right|}\underset{y\in S_{r}(x)}{\sum }f\left(y\right),$$

where |*S*_*r*_(**x**)| is the number of pixels in *S*_*r*_(**x**) and *f*(**y**) is the image intensity at pixel **y**. 

The *variance* at pixel **x** is defined as

(6) $$\sigma^{2}\left(x\right)=\frac{1}{\left|S_{r}\left(x\right)\right|-1}\underset{y\in S_{r}(x)}{\sum }{\left[f\left(y\right)-\mu \left(x\right)\right]}^{2},\;\left|S_{r}\left(x\right)\right|>1.$$

Variance may only be calculated when |*S*_*r*_(**x**)| > 1, since when *r*(**x**) = 0 there is only one pixel in *S*_*r*_(**x**) and variance is not defined. The standard deviation *σ*(**x**) of the intensities of the set of pixels within the sphere centered at pixel **x** is simply the square root of the variance.

The *first-order moment* of intensities within *S*_*r*_(**x**) is given by

(7) $$m\left(x\right)=\underset{y\in S_{r}(x)}{\sum }\left(y-x\right)f\left(y\right).$$

Due to the finite extent of an image's domain *Ω*, a sphere may be truncated by one or more edges of the image (e.g, *S*_3_(**x**) in Figure 2). Unlike conventional kernels, which usually require pixel values outside the image to be arbitrarily defined, our spherical sets simply exclude such locations from calculations. Thus, truncation will not adversely affect *μ*(**x**) or *σ*(**x**), other than by reducing the sample size; it will not bias the result by introducing some arbitrary values for pixels outside the image. The first-order moment of a truncated sphere, however, does exhibit an edge effect, due to its asymmetrical pixel distribution. We compensate for this by defining a measure, *VSS gradient*, which corrects the first-order moment of intensities to remove the edge effect. Given the center of mass of pixel locations in sphere *S*_*r*_(**x**),

(8) $$c\left(x\right)=\frac{1}{\left|S_{r}\left(x\right)\right|}\underset{y\in S_{r}(x)}{\sum }y,$$

the VSS gradient ∇*f*(**x**) is defined as

(9) $$\nabla f\left(x\right)=\frac{1}{\left|S_{r}\left(x\right)\right|}\left[m\left(x\right)-\mu \left(x\right)\left(c\left(x\right)-x\right)\right].$$

**Figure 4:**
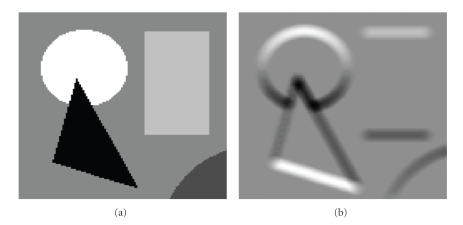
(a) Synthetic noiseless image. (b) Corresponding vertical component of the VSS gradient (radius = 3 for all spheres). Note there are no edge effects, such as those seen with conventional gradient calculations.

Note that for nontruncated spheres **c**(**x**) = **x** and VSS gradient is equivalent to the moment vector normalized to the number of pixels, **m**(**x**)/|*S*_*r*_(**x**)|. For truncated spheres, the VSS gradient does not suffer from the edge effects typical of convolution kernels used to measure gradient (see Figure 4). 

All of the above *primary statistics* depend only on the pixels within a given sphere. As a sphere grows or shrinks, its primary statistics can be computed incrementally by adding or removing shells, significantly reducing computational load during sphere map optimization.

## 5. Secondary Statistics

We define *secondary statistics* as higher order VSS derived by combining multiple spheres to perform more complex analyses of neighborhoods. One such neighborhood that is useful in these computations,

(10) $$S^{-1}\left(x\right)=\left\{y:x\in S_{r}\left(y\right)\right\},$$

is the set of all pixels whose spheres contain **x**. The −1 superscript in *S* ^−1^(**x**) is used to impart the flavor of an inverse function. Since spheres adjust their radii individually, the number of spheres that contain a given pixel varies widely. However, since it is always true that **x** ∈ *S*_*r*_(**x**), it is also always true that **x** ∈ *S* ^−1^(**x**); every pixel is contained by its own sphere.

**Figure 5:**
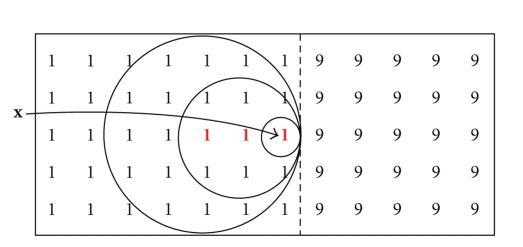
The *S* ^−1^(**x**) set of spheres that contain pixel **x**, adjacent to the boundary between two noiseless regions (same as Figure 2) with respective intensities of 1 and 9.

Given a correctly optimized sphere map, *S* ^−1^(**x**) will consist entirely of pixels from the same object as pixel **x**. Figure 5 shows members of one such *S* ^−1^(**x**) set, consisting of three pixels (bold), whose spheres each contain **x**. Notice that all three spheres touch but do not cross the boundary, so they are correctly optimized. (There would be other spheres as well in a correctly optimized *S* ^−1^(**x**).)

Secondary statistics are derived from populations of spheres such as *S* ^−1^(**x**). Thus, *μ*_*μ*_(**x**), the *mean of means* at pixel **x** is defined as

(11) $$\mu_{\mu }\left(x\right)=\frac{1}{\left|S^{-1}\left(x\right)\right|}\underset{y\in S^{-1}(x)}{\sum }\mu \left(y\right),$$

which is the mean of the mean intensities for all the spheres in *S* ^−1^(**x**). In a noiseless image containing distinct homogeneous regions, the correctly optimized sphere map yields values for *μ*_*μ*_(**x**) identical to the original intensity image *f*(**x**). In a noisy image, as will be shown, *μ*_*μ*_(**x**) yields a an image with reduced noise but with sharp boundaries. 

Likewise, *σ*_*μ*_(**x**), the standard deviation of the mean intensities for the spheres in *S* ^−1^(**x**) is defined as

(12) $$\begin{array}{cc} & \sigma_{\mu }\left(x\right)\\ & ={\left[\frac{1}{\left|S^{-1}\left(x\right)\right|-1}\underset{y\in S^{-1}\left(x\right)}{\sum }{\left[\mu \left(y\right)-\mu_{\mu }\left(x\right)\right]}^{2}\right]}^{\frac{1}{2}},\left|S^{-1}\left(x\right)\right|>1. \end{array}$$

Note that, as with *σ*(**x**), the above definition of *σ*_*μ*_(**x**) is given only for |*S* ^−1^(**x**)| > 1, since at least two samples are required for variance or standard deviation to be meaningful. For noiseless images, a correct sphere map will yield a value of zero for *σ*_*μ*_(**x**) at every pixel.

**Figure 6:**
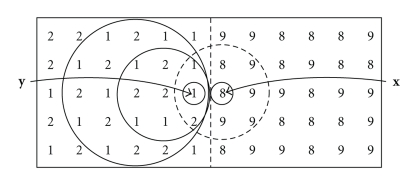
Image with noise. Pixel **x** is deterred from extending its sphere across the boundary because its mean is an outlier in the population *S* ^−1^(**y**).

We can now compute *z*_*μ*_(**x** | **y**), the *z-value* for the sphere **x** to belong to the set of spheres that already include **y**. We define *z*_*μ*_(**x** | **y**) as

(13) $$z_{\mu }\left(x\;|\;y\right)=\frac{\left|\mu \left(x\right)-\mu_{\mu }\left(y\right)\right|}{\sigma_{\mu }\left(y\right)},$$

to provide a measure of how well *S*_*r*_(**x**) fits into the current *S* ^−1^(**y**) set. The justification is that in an optimized sphere map, if *S*_*r*_(**x**) were to justifiably contain pixel **y**, then *μ*(**x**) should fall within the distribution of means for all spheres that already contain **y**. This concept is illustrated in Figure 6, which shows pixel **x** attempting to extend its sphere across the boundary to include pixel **y**. We have included noise in the image to demonstrate that a high *z*-value could be used to stop the growth of *S*_*r*_(**x**) at the boundary, even in the presence of such noise. It should be noted that the utility of this statistic is dependent on a reasonable initialization of the sphere map, such that the percentage of spheres already not crossing boundaries is high enough to lend statistical validity to *μ*_*μ*_(**y**) and *σ*_*μ*_(**y**).

## 6. Noiseless Sphere Map Optimization Algorithm

Armed with the primary and secondary statistics just described, we are ready to develop algorithms to optimize a sphere map, that is, to set the correct radius of each sphere *S*(**x**) in an image such that it reaches, but does not cross, the nearest boundary. We will develop two such algorithms here. The first is designed to optimize a sphere map on a noiseless image, a far simpler task than optimizing a sphere map for a real image.

**Figure 7:**
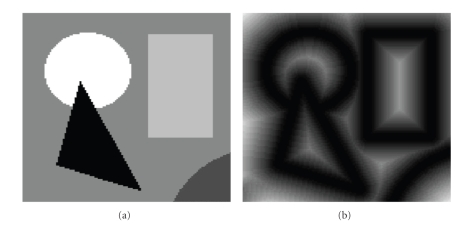
(a) An example noiseless image. (b) The sphere map created by the noiseless algorithm, represented as an image where each pixel **x** has an intensity value equivalent to the radius of its sphere *S*(**x**).

A noiseless image consists of a set of objects, each containing a set of pixels of uniform intensity. Figure 7(a) shows an example of a synthetically generated noiseless image. Object boundaries can easily be determined in such images by detecting any change in intensity between pixels. Our noiseless algorithm functions simply by growing each sphere *S*(**x**) in the image until it contains a pixel **y** in its next outer shell, **y** ∈ *H*_*r*+1_(**x**), with a different intensity value than that of pixel **x**, resulting in a nonzero variance *σ* ^2^(**x**). This process produces a sphere map containing the correct radii of all spheres within the image, shown in Figure 7(b), whose intensity represents *r*(**x**). Notice the bright ridges within and between objects, representing medial ridges, where spheres touch at least two boundary points. The noiseless algorithm is useful for finding medial ridges in cases where an unambiguous segmentation has already been obtained. We have used it, for example, on manual segmentations of the amygdala in the magnetic resonance (MR) images of the human brain, to determine medial parameters of shape for diagnosis of depression [14, 15].

## 7. Real Image Segmentation Algorithm

Real images contain noise and therefore require greater finesse and complexity in establishing the optimal sphere map. The Shells and Spheres framework lends itself to the design of many different algorithms for this purpose. We present here in detail one such algorithm that was the subject of the masters and doctoral dissertations of [16, 17]. 

The algorithm takes the form of a six-step process, with Steps 1–4 optimizing the sphere map, Step 5 finding medial pixels, and Step 6 producing a segmentation of the target object. Step 1 uses VSS gradient to detect boundaries and creates an initial approximation of the correct sphere map, from which acceptable statistical values can be obtained for use in subsequent steps. Step 2 utilizes the pronounced discrepancy in pixel variance between spheres that have incorrectly grown across boundaries and those that have not, to reduce the size of incorrect spheres, placing them correctly within their appropriate image objects. Step 3 introduces specialized boundary indicators, known as *outposts*, designed to stop spheres from crossing into a new image object, using information extracted from population testing between spheres in adjacent objects. These outposts influence the radii of nearby spheres, resizing them to adhere to a consensus on boundary location. Step 4 revisits variance calculation using the current sphere map, which is more accurate than the sphere map previously available. The new variance measure is applied to spheres, encouraging them to fully grow within their respective image objects, effectively smoothing the radius image and sharpening its boundaries (as defined by all spheres of radius 0 or 1). Step 5 identifies medial pixels, that is, those whose spheres touch at least two boundaries. Given a seed point within the target object, Step 6 locates the nearest medial pixel and connects neighboring medial pixels, combining their corresponding spheres to produce a segmentation. The following sections describe each step in detail.

**Step 1 (VSS Gradient-Based Radius Approximation)**

**Figure 8:**
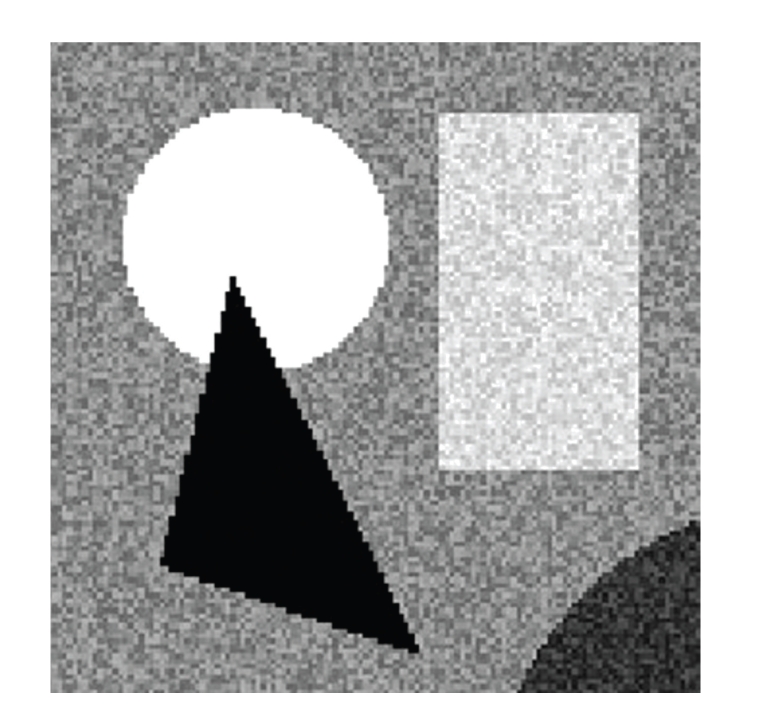
Synthetic test image from Figure 7(a) with gaussian noise added.

As previously illustrated, it is trivial to optimize the sphere map for a noiseless image by growing spheres until a new intensity value is detected. When analyzing real images, however, this approach will fail, because intensity variation due to noise may be indistinguishable from an object boundary, especially within small spheres. For illustrative purposes, we consider the previous synthetic image with Gaussian noise added, shown in Figure 8.

Steps 2, 3, and 4 depend on there already existing a sphere map that is at least somewhat accurate, because those steps make use of secondary VSS, based on collections of existing spheres. For Step 1 to accomplish this first attempt at an accurate sphere map, all spheres are first set to *r*(**x**) = 0 and are then allowed to grow until a persistent increase in VSS gradient magnitude (9) is detected over a continuous range of sphere sizes. Unlike conventional gradient measured with a fixed-scale kernel, the VSS gradient depends locally on *r*(**x**) and is based on the first-order moment of intensity normalized by the number of pixels in the sphere. Thus the VSS gradient can be expected to increase monotonically as a sphere grows past a boundary, since the first-order moment favors the outer pixels. A persistent increase in VSS gradient for *g* consecutive steps is sought, indicating that a boundary has been crossed, whereupon *r*(**x**) is reset to the radius just before the increase in VSS gradient began. Empirically, it has been found that a value of *g* = 5 performs well for both MRI and CT cardiac data to achieve a reasonable first approximation of the optimized sphere map. We found Step 1 to be effective at growing spheres past tissue inhomogeneity and noise. However, spheres may not stop exactly on the boundary because of the effect of noise on the detection of consistent gradient increase. Using VSS gradient to govern sphere growth can also fail completely for a sphere that encounters two opposing boundaries simultaneously, as their contributions to the gradient may cancel.

**Step 2 (Variance-Constrained Radius Reduction)**

After Step 1, three possible states exist for each sphere: The sphere can be too large, too small, or the correct size (i.e., it touches the nearest boundary but does not cross it). The most glaring error in the sphere map after Step 1 is the presence of large-scale spheres that have incorrectly grown past boundaries. As previously mentioned, this type of error typically occurs when a growing sphere contacts multiple boundaries at once, which indicates that the sphere lies on the medial manifold (the locus of centers of spheres lying within an object that touch at least two boundaries). In such a case, the contributions to the VSS gradient from multiple boundaries may cancel, allowing the sphere to grow much larger than its correct radius. Such spheres, luckily, have variance values that are orders of magnitude higher than those that remain within a single object.

**Figure 9:**
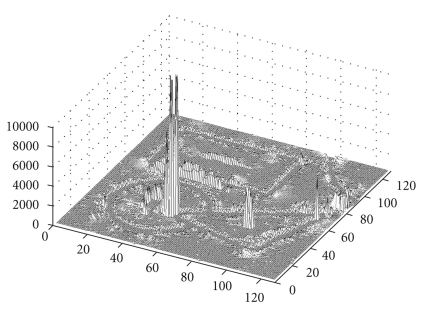
A height map of the variance *σ* ^2^(**x**) after Step 1 is applied to the image in Figure 8, showing large spikes where spheres encounter two boundaries at once, canceling VSS gradient and growing too large.

Figure 9 shows a height map of variance values *σ* ^2^(**x**) for all spheres after applying Step 1 to the synthetic image with added noise shown in Figure 8. The flat “floor” section of the height map is the variance of the uniform noise added to the image, which has a value near 200. Spheres crossing boundaries, however, have a much larger variance than spheres that correctly remain within the object boundaries, extending to a value of nearly 16000. This large dynamic range can be exploited to shrink the spheres that grow too large. All spheres with a variance above a certain threshold *α*_*s*_ are shrunk by decrementing *r*(**x**) until *σ* ^2^(**x**) < *α*_*s*_. (The subscript “*s*” stands for *shrink*.) The threshold *α*_*s*_ is defined as

(14) $$\alpha_{s}=\mu_{\sigma^{2}}+\beta_{s}\sigma_{\sigma^{2}},$$

where *μ*_*σ*^2^_ and *σ*_*σ*^2^_ are the mean and standard deviation, respectively, of the variance of all the spheres in the current sphere map. The positive constant *β*_*s*_ thus represents the number of standard deviations above the mean permitted for a sphere's variance without the sphere being required to shrink. The exact value of this parameter is not particularly critical, as the difference between the spheres that grew much too large and the others is very large. We found typical value for *β*_*s*_ to be 0.2, although as will be described below the value was optimized for individual data sets. Reducing the radius of pixels with extremely high variance corrects a majority of the spheres that have incorrectly grown past boundaries.This global threshold for *σ* ^2^(**x**) is not ideal, because it assumes a constant expected variance throughout the image. This expectation is likely untrue, given factors such as tissue inhomogeneity and nonuniform noise, and ongoing work is exploring ways around this problem. It should also be noted that *σ* ^2^(**x**), and thus the threshold *α*_*s*_, depend on the current sphere map, *r*(**x**), which is not yet fully optimized at this step. This deficiency is addressed by returning to variance in Step 4, once a more accurate *r*(**x**) is available.

**Step 3 (Reflectance and Outposts)**

Following Step 2, many spheres are correctly sized and face each other across boundaries. This sets the stage for the use of secondary VSS to differentiate regions on opposite sides of those boundaries. Likely boundary candidates are identified for a given sphere at location **x** by finding a pixel **y** in its *H*_*r*+1_(**x**) shell with a high value for *z*_*μ*_(**x** | **y**). As already discussed, and illustrated in Figure 6, such a sphere will detect growth past a boundary by finding itself unlike the *S* ^−1^(**y**) population of spheres containing the pixel **y** just across the boundary. The sphere at **x** is said to place a *reflector* at such a location, a metaphorical construct denoting a vote by the sphere at **x** for the pixel **y** as being across the nearest boundary. Note that we do not vote for pixel **y** as a boundary itself. Instead, pixels on each side of a detected boundary are marked by each other as being accros the boundary. Thus, referring again to Figure 6, *S*_*r*_(**x**) could place a reflector at pixel **y**. The set of reflectors placed by a given sphere *S*_*r*_(**x**) is denoted, *K*(**x**). In the present algorithm the constraint |*K*(**x**)| = 1 is applied, limiting each sphere to placing only one reflector, for reasons discussed below. This constraint leads to the definition of *K*(**x**) as

(15) $$K\left(x\right)=\left\{y:y=\underset{y\in H_{r+1^{\left(x\right)}}}{\text{argmax}}\;\;\;z_{\mu }\left(x\;|\;y\right)\right\}.$$

**Figure 10:**
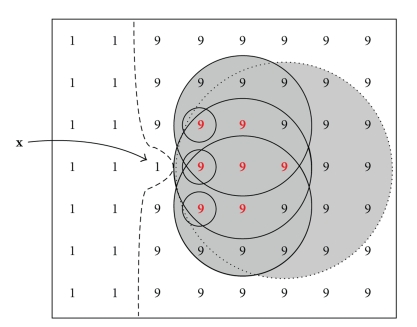
Illustration of *K* ^−1^(**x**) containing 7 pixels (bold), each of whose spheres would place its reflector across the boundary at **x**.

**Figure 11:**
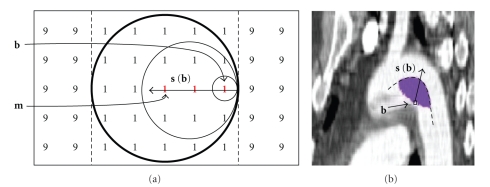
(a) Diagram of an object with intensity 1 between two regions of intensity 9, showing set *S* ^−1^(**b**) of pixels (bold) whose spheres contain pixel **b**. This set produces an **s**(**b**) vector (see text) along which the furthest bold pixel **m** is the center of a medial sphere (circle in bold) touching both boundaries (dashed lines). (b) Image of a 2D slice through a CT scan of the aorta with contrast showing an actual *S* ^−1^(**b**) set (purple), the resulting **s**(**b**) vector, and the medial manifold (dashed curve) on which the furthest sphere along **s**(**b**) must lie.

If a boundary exists just beyond the outer shell of *S*_*r*_(**x**), it will be located at the pixel **y** for which the highest *z*_*μ*_(**x** | **y**) is calculated. If multiple object boundaries exist just beyond the outer shell of *S*_*r*_(**x**), the boundary producing the largest *z*_*μ*_(**x** | **y**) will be marked with a reflector.Each pixel may contain reflectors placed by a number of spheres. The set of spheres that have placed reflectors at a pixel **x** is defined as

(16) $$K^{-1}\left(x\right)=\left\{y:x\in K\left(y\right)\right\},$$

invoking the same inverse notation used for *S* ^−1^(**x**) in (10). A set of spheres placing their reflectors across a boundary at pixel **x** is shown in Figure 11. The number of reflectors |*K* ^−1^(**x**)|  at a given pixel is referred to as the *reflector count*. For example, in Figure 10, the reflector count |*K* ^−1^(**x**)| = 7. A reflector placed by **y** at **x** has an inherent direction governed by the vector (**y** − **x**). The vector sum of the directions of all of the reflectors at **x** is denoted, the *reflectance* **k**(**x**), defined by

(17) $$k\left(x\right)=\frac{1}{\left|{\text{K}}^{-1}\left(x\right)\right|}\underset{y\in K^{-1}(x)}{\sum }\frac{y-x}{\left|y-x\right|}.$$

This measure provides the average orientation of the *K* ^−1^(**x**) population, which describes a direction generally normal to the boundary, pointing to the center of the region represented by *K* ^−1^(**x**). Since it was decided that each sphere will contribute exactly one reflector, reflector density and reflectance are normalized over the image. Therefore, reflector count can be used to differentiate between significant collections of reflectors correctly placed at boundaries and sparse distributions of reflectors incorrectly placed in the interior of objects. To denote pixels containing a significant number of reflectors, the term *outpost* is adopted, since such pixels serve as border markers and face each other across boundaries much the same way that military outposts of opposing armies face each other across the battle line.The set of all pixels in an image chosen to be outposts is denoted by *P*. In the present algorithm this set is found in two steps. First, the set of *primary outposts* *P*′ is established, containing all pixels with zero radius and at least *κ* reflectors, that is,

(18) $$P^{\prime }=\left\{x:\left|K^{-1}\left(x\right)\right|\geq \kappa ,\;\;r\left(x\right)=0\right\}.$$

For the results presented in this paper, *κ* = 4. To increase the density of outposts along the boundaries, a set of *secondary outposts* *P*′′ is generated, containing all pixels with zero radius that adjoin an outpost in *P*′ and have at least *λ* reflectors, where *λ* < *κ*,

(19) $$P^{\prime \prime }=\left\{x:\left|K^{-1}\left(x\right)\right|\geq \lambda ,{\;\;H}_{1}\left(x\right)\cap P^{\prime }\neq \emptyset ,\;\;r\left(x\right)=0\right\}.$$

For the results presented, *λ* = 2. By combining the sets of primary and secondary outposts, the set of all outposts,

(20) $$P=P^{\prime }\cup P^{\prime \prime }$$

is formed.Each outpost **y** ∈ *P* has a reflectance **k**(**y**). A sphere at **x** can distinguish whether a given outpost is on its side of the boundary, constituting a *friendly outpost*, or the other side of the boundary, constituting an *enemy outpost*, based on the direction of the outpost's reflectance. The set of enemy outposts (those with reflectance facing **x**) within the sphere of radius *r*(**x**) is defined as

(21) $$E_{r}\left(x\right)=\left\{y:y\in P\cap S_{r}\left(x\right),\;\;k\left(y\right)\cdot \left(y-x\right)<0\right\},$$

where the sign of the dot product determines the direction of **k**(**y**) relative to **x**.In governing the growth of a sphere, enemy outposts are to be avoided, while friendly outposts can be included. More specifically, enemy outposts should stop the growth of spheres, as they represent a different image region than the one in which the sphere resides, while friendly outposts do not. Step 3 uses the number of enemy outposts to adjust the sphere size as follows: If the pixel contains no enemy outposts in its next shell out, *H*_*r*+1_(**x**), the sphere grows until it does. That is,

(22) $$\text{if}\;\;\;\left|E_{r+1}\left(x\right)\right|=0,\;\text{increase}\;r\left(x\right)\;\text{until}\;\left|E_{r+1}\left(x\right)\right|>0.$$

If the number of enemy outposts included in the set *S*_*r*_(**x**) is greater than *γ*, the radius is decreased until this is no longer true, that is, 

(23) $$\text{if}\;\;\;\left|E_{r+1}\left(x\right)\right|>\gamma ,\;\;\text{reduce}\;\;\;r\left(x\right)\;\text{until}\;\left|E_{r}\left(x\right)\right|\leq \gamma .$$

In the present implementation, *γ* = 2. This value prevents lone pixels that have been improperly labeled as outposts from incorrectly causing spheres to shrink.After Step 2, in which incorrectly large spheres have been adjusted to a more correct size, the most pressing problem with the sphere map is the scattered effects of noise on *r*(**x**). Step 3 focuses on spheres that have incorrectly stopped growth at image noise, or grown slightly too large across their nearest boundary. The effect of these outpost-driven operations is that significant densities of reflectors placed by correctly-sized spheres along boundaries are used to govern the size of other spheres, sweeping incorrect reflectors from the within objects to the boundaries (since a sphere's reflector is redistributed when its radius is altered). Since each sphere places only one reflector, some pixels along object boundaries may remain unmarked, leading to a somewhat sparse collection of outposts along boundaries. This will not adversely effect the evolution of the sphere map, however, due to another advantage of our spherical operator design. Because most spheres are large relative to the spacing of outposts along the boundary, their growth will be stopped and they tend not to “leak” or “bleed” across boundaries, as some conventional deformable contours are prone to do.

**Step 4 (Variance-Constrained Scale Growth)**

At this point in the analysis, our sphere map has achieved a configuration generally representative of the shapes within the image, but it still retains adverse effects from noise and suboptimal boundary detection. Although Step 3 results in a reasonably accurate *r*(**x**), some spheres still may not quite reach boundaries, due to pixels being incorrectly labeled as outposts. These false outposts will stop spheres in the interior of image objects, leading to potential errors in segmentation. As an added measure to force spheres to grow maximally within their objects, we return to variance a second time, calculated in the same manner as in Step 2, but used to grown now rather than to shrink.A second global variance threshold for variance, *α*_*g*_, similar to *α*_*s*_ described above, is calculated as

(24) $$\alpha_{g}=\mu_{\sigma^{2}}+\beta_{g}\sigma_{\sigma^{2}},$$

where *μ*_*σ*^2^_ and *σ*_*σ*^2^_ again are the mean and standard deviation, respectively, of the variance throughout the image (the subscript “*g*” stands for *grow*). The threshold *α*_*g*_ is used to smooth the boundaries in the radius image by forcing spheres to grow up to the actual boundary using the current, more accurate variance *σ* ^2^(**x**). The value of *r*(**x**) is incremented for all spheres while their internal variance *σ* ^2^(**x**) < *α*_*g*_. This creates a sphere map that more accurately matches the contours of objects in the image.At this point the sphere map is considered optimized.

**Step 5 (Medial Pixel Identification)**

Given the optimized sphere map *r*(**x**), the next goal is to extract medial pixels. To facilitate this, a dense set of boundary pixels *B* is first defined, as those whose spheres have radius 0 or 1,

(25) B={x:r(x)≤1}.

The sets *S* ^−1^(**b**) for all boundary pixels **b** ∈ *B* can be used to find pixels on the medial manifold, whose spheres touch two boundaries while still lying completely within the object. Recall that the *S* ^−1^(**b**) set for pixel **b** contains all spheres in the sphere map that themselves contain pixel **b** (10). Given a correct sphere map, this set will necessarily contain at least one sphere that touches both the boundary that pixel **b** borders as well as an opposing boundary across the object region (and also across the sphere) from pixel **b**. Figure 11(a) shows such a medial pixel (labeled “*m*”) on the medial manifold of an object of intensity 1, between two regions of intensity 9.To find such medial pixels within *S* ^−1^(**b**), we first define an orientation **s**(**b**), roughly orthogonal to the boundary, as the vector sum of the normalized offsets relative to **b** for pixels within *S* ^−1^(**b**) as

(26) $$s\left(b\right)=\frac{1}{\left|S^{-1}\left(b\right)\right|}\underset{y\in S^{-1}(b)}{\sum }\frac{y-b}{\left|y-b\right|}.$$

**Figure 12:**
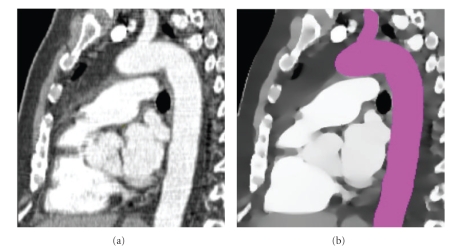
(a) Two-dimensional sagital slice through computer tomography (CT) data of thorax with vascular contrast used in preliminary testing of our algorithm. (b) Mean of means *μ*_*μ*_(**x**) image showing reduced noise and sharp boundaries, automated segmentation of aorta highlighted (purple) created from a single-seed point.

For each boundary pixel **b** ∈ *B*, the pixel **m** ∈ *S* ^−1^(**b**) that is furthest from the boundary along **s**(**b**) is identified as a medial pixel, as depicted in Figure 12(a). The set of all medial pixels *M* in the image is thus

(27) $$M=\left\{m:m=\underset{y\in S^{-1}\left(b\right)}{\text{argmax}}\;\left(\left(y-b\right)\cdot s\left(b\right)\right),\;\;b\in B\right\}.$$

Figure 11(b) shows an actual *S* ^−1^(**b**) set for a pixel **b** on the boundary of the aorta in a 2D slice through a computed tomography (CT) scan with contrast. The furthest pixel along vector **s**(**b**) lies on that medial manifold (dashed line).Selecting a single pixel from each *S* ^−1^(**b**) set overlooks a potentially large number of additional medial pixels on the outer edge of each set, especially for a concave boundary point such as shown in Figure 12. One can, however, be certain that each *S* ^−1^(**b**) set contains a minimum of one medial pixel, as the center of the largest sphere in the direction roughly orthogonal to the boundary. The set *M* derived taking advantage of this fact is a sparse but reliable set of pixels on the various medial manifolds within the image.

**Step 6 (Medial Flood-Fill Segmentation)**

To segment a particular object, a seed pixel **p** ∈ *M* on that object's medial manifold is needed. To find it, a sample pixel is manually selected by the user, and a search is conducted for the closest medial pixel by iterating through successive shells moving radially outward from the selected pixel. The first medial pixel encountered is accepted as **p**. A flood fill operator is then used to find a connected subset *C*⊆*M*. Pixels belonging in *C* are found iteratively using a series of sets *C*_*i*_ starting with *C*_0_, a set containing just the seed pixel **p**. At each subsequent step *i* + 1, the set *C*_*i*+1_ is created by adding medial pixels within a radius *m* of pixels already in set *C*_*i*_. More precisely, *C*_*i*_ is defined recursively as

(28) $$\begin{array}{c} C_{0}=\left\{p\right\},\\ C_{i+1}\left\{x:x\in M,\;\;S_{m}\left(x\right)\cap C_{i}\neq \emptyset \right\}. \end{array}$$

For the results presented, radius *m* was dynamically set to *m*(**x**) = *r*(**x**)/2 as this causes the algorithm to search halfway from the medial manifold to the boundary for new medial pixels to include, therefore staying within the designated object. When a final step *f* adds no new pixels, such that

(29) $$C_{f}=C_{f-1},$$

the flood-fill is complete, as the set of connected medial pixels within the object is the current pixel collection, or

(30) $$C=C_{f}.$$

The union of the set of spheres centered at these medial pixels effectively segments the object by including all of the pixels designated as within the object. These spheres, centered on the medial manifold, extend to all points on the boundary.

## 8. Results

The present paper is the first disclosure in a journal article of the detailed mathematics of Shells and Spheres. Validation of the particular algorithm just described has already been published [18], so we only summarize those results briefly here. Figure 12 shows a segmentation of the aorta on a 2D slice through a throracic CT scan with contrast. On the left, the raw CT data is shown to have considerable noise. On the right, the mean of means image *μ*_*μ*_(**x**) exhibits greatly reduced noise while maintaining sharp boundaries and significant detail. The aorta has been segmented (purple) by initializing a flood-fill operation with a single-seed point near its medial manifold.

**Figure 13:**
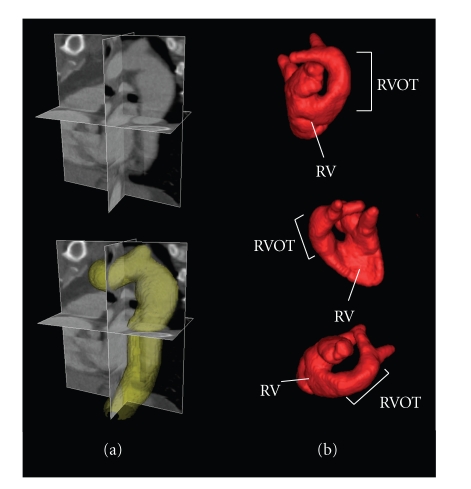
(a) 3D contrast-enhanced CT scan of the aorta (top left), and the same scan with an overlaid 3D segmentation (bottom left) achieved using the Shells and Spheres framework. (b) Surface rendering of a Shells and Spheres segmentation of the right heart with labeled Right Ventricle (RV) and Right Ventricular Outflow Tract (RVOT), shown from three different perspectives.

Figure 13(a) shows a 3D segmentation of the aorta using the same algorithm (which is inherently *n*-dimensional). The top portion shows the raw CT data and the bottom shows a surface rendering of the segmented aorta as the union of all the medial spheres. 

Figure 13(b) shows the results of segmenting the heart in 3D magnetic resonance (MR) data. In this case, we performed parameter optimization using a single manual tracing on a 2D slice selected from a 3D image data set, to find optimum values for *β*_*s*_ and *β*_*g*_, used to compute the thresholds for variance-based shrinking and growing in Steps 2 and 4 of the algorithm, respectively. Otherwise, the algorithm is completely automatic. 

**Figure 14:**
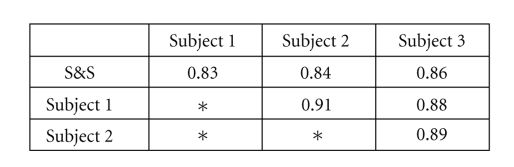
Table of DSC values comparing segmentations produced by 3 independent subjects and our Shells and Spheres algorithm.

To test the accuracy of the 3D segmentation, a validation study was conducted to compare it to three manual segmentations. Each segmentation was produced by a different user. Figure 14 shows the Dice Similarity Coefficient (DSC) values for our automated segmentation compared to the manual segmentations, as well as the DSC values for the manual segmentations compared to each other. The DSC produces the value 1 for identical segmentation and 0 for segmentations that do not overlap at all. It can be seen that our automated segmentation matches the manual segmentations with a DSC between 0.83 and 0.86. It should be noted that a DSC of 0.70 is considered excellent agreement in the literature [19], although the definition of sufficient accuracy is, of course, specific to the application. While the manual segmentations produced slightly higher agreement with each other than with the automated segmentation, it is believed that a significant portion of this discrepancy is due to the difficulty for the algorithm in defining the extent of the “right heart” along the continuum of the circulatory system, rather than the boundaries of the vessels themselves. Subject 3 elected to include less of the branching vasculature connected to the main cardiac structures, which led to greater agreement with the automated segmentation and less agreement with the other manual segmentations. Despite the variation in manual segmentations, our system still demonstrated reliable segmentation results.

Comparison with other segmentation techniques is always desirable, but problematic in view of the enormous number of methods that have been developed. We compared our method against two other techniques, an active contour method and intesity thresholding. We present those results in the following section.

**Figure 15:**
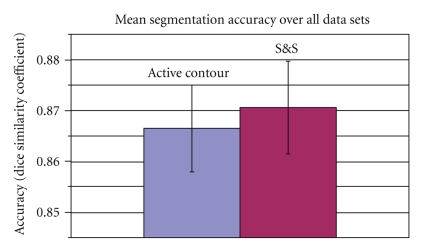
Bar graph showing the mean 3D segmentation agreement to all expert segmentations over all data sets for both ITK-SNAP active contour method (blue) and the Shells and Spheres method (maroon). Error bars show the standard error of the mean (SEM).

Segmentations of the RVOT in our 3D ovine MRI data sets were produced with a widely used geodesic active contour method implemented in the Insight Toolkit ITK-SNAP software package [20]. As before, the DSC was used to show agreement to the manual 3D segmentations of the RVOT produced by our three experts. Results of comparing the active contour method to our Shells and Spheres method can be seen in Figure 15.

This graph shows the mean segmentation agreement to all expert segmentations over ten ovine MRI data sets for the active contour method and our Shells and Spheres method. The error bars were determined by the Standard Error of the Mean (SEM). 

We can see that the Shells and Spheres segmentation system performed slightly better than the active contour method, but the high degree of overlap of the respective SEM values indicates roughly equivalent performance. An independent-samples *t*-test showed that the two means were not significantly different (*P*-value  =  .741). We conclude that Shells and Spheres can match this current clinical state of the art-automated segmentation method. Furthermore, the minimal manual input required by the Shells and Spheres algorithm at the onset of analysis (tracing a single 2D slice for parameter optimization) represents considerable less time and effort on the part of the human operator than the continual supervision necessary for the active contour method. Additionally, our system is designed to require only skills and expertise inherent to the clinical professional, rather than expecting a medical professional to gain algorithmic or mathematical expertise to effectively perform the active contour segmentation.

Intensity thresholding coupled with a flood-fill from a manually placed seed point was also explored as a common technique for comparison to our segmentation system, but the prevalence of partial-volume effects and tissue inhomogeneity in MRI images made this method incapable of segmenting the RVOT in our data sets, due to bleeding of the floodfill regardless of threshold parameters. Without a high degree of manual postprocessing, this method produced a failed segmentation (DSC < 0.70 for all expert segmentations) on each of our MRI data sets.

## 9. Discussion

The Shells and Spheres framework for image analysis and the associated *n*-dimensional algorithms described here represent a novel system to facilitate image segmentation. Advantages include preservation of sharp boundaries while including large populations of pixels from both sides of the boundaries for statistical analysis. The primary statistics exhibit no edge effect and can be efficiently computed by adding and subtracting shells while optimizing the sphere map. Because the framework is truly *n*-dimensional, volumetric segmentation occurs in 3D, not slice by slice, lending a natural anatomical appearance to the visualized surface. Finally, the algorithm presented yields useful medial features for further analysis, which is highly relevant to understanding anatomical shape. 

We have presented just two example algorithms. Many others are possible. One that is currently under development introduces a new secondary statistic, a variation on the Student's *t*-test, incorporating mean and variance in a way that further sharpens boundary detection. Subsequent determination of the medial manifold uses the divergence of the unit direction to the nearest boundary [21], an adaptation of concepts recently developed by Dimitrov et al. [22] which are particularly robust in the presence of noise and efficient to compute when incorporated into the Shells and Spheres framework.
